# Author Correction: A protocol for adding knowledge to Wikidata: aligning resources on human coronaviruses

**DOI:** 10.1186/s12915-023-01764-2

**Published:** 2023-11-16

**Authors:** Andra Waagmeester, Egon L. Willighagen, Andrew I. Su, Martina Kutmon, Jose Emilio Labra Gayo, Daniel Fernández-Álvarez, Quentin Groom, Peter J. Schaap, Lisa M. Verhagen, Jasper J. Koehorst

**Affiliations:** 1 Micelio, Antwerp, Belgium; 2 NUTRIM, Maastricht University, Maastricht, The Netherlands; 3 Department of Integrative Structural and Computational Biology, The Scripps Research Institute, La Jolla, CA USA; 4 Maastricht Centre for Systems Biology (MaCSBio), Maastricht University, Maastricht, The Netherlands; 5 WESO Research Group, University of Oviedo, Oviedo, Spain; 6 Meise Botanic Garden, Meise, Belgium; 7 Department of Agrotechnology and Food Sciences, Laboratory of Systems and Synthetic Biology, Wageningen University & Research, Wageningen, The Netherlands; 8 Intravacc, PO Box 450, 3720 AL Bilthoven, The Netherlands

**Correction: BMC Biol 19, 12 (2021)**

**https://doi.org/10.1186/s12915-020-00940-y**

The original article [1] contains an incorrect version of Fig. 3. The corrected version can be viewed ahead in this Correction article.

**Fig. 3:**
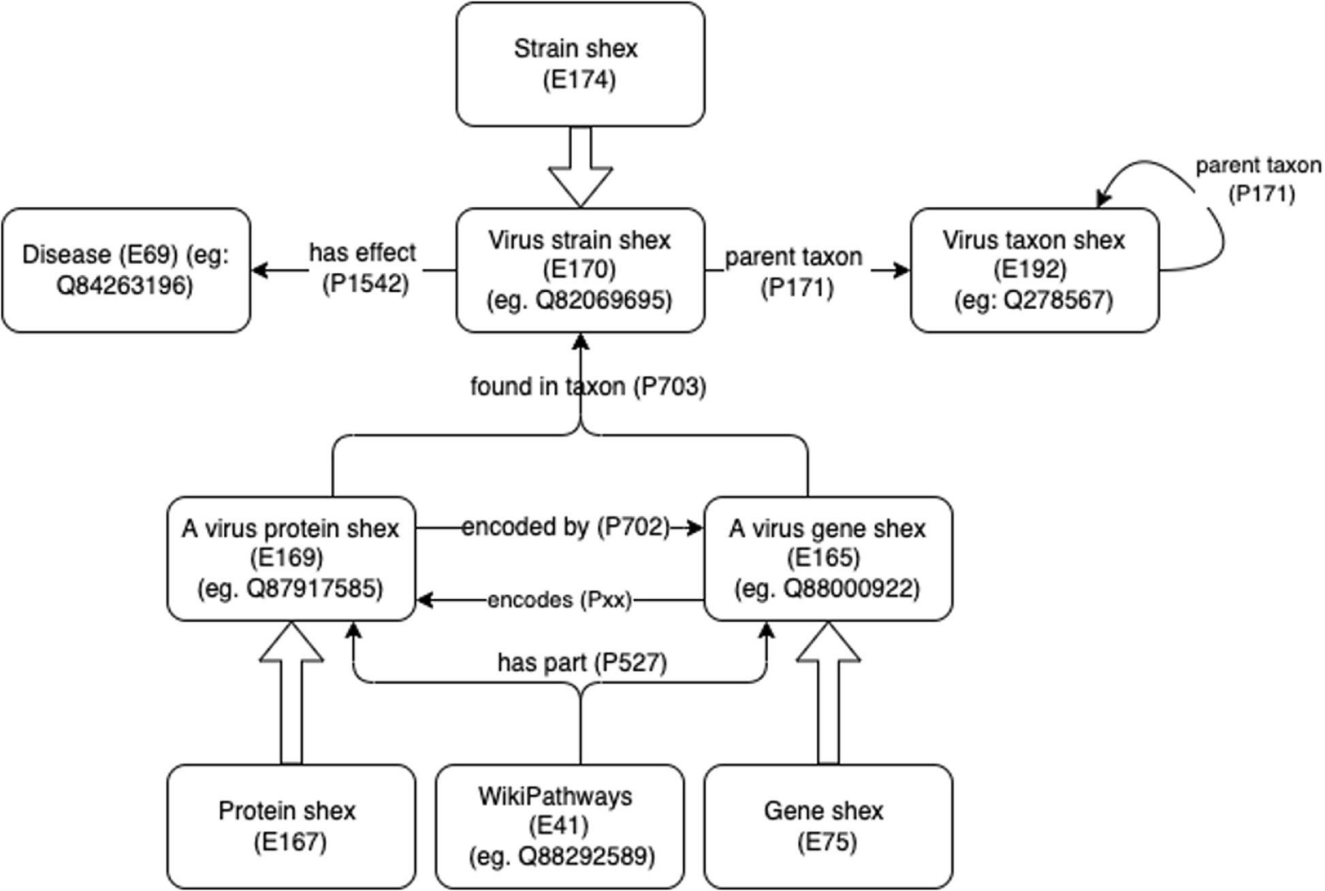
Overview of the ShEx schemas and the relations between them. All shapes, properties, and items are available from within Wikidata
